# Lignin as a Renewable Building Block for Sustainable Polyurethanes

**DOI:** 10.3390/ma15176182

**Published:** 2022-09-05

**Authors:** Fernanda Rosa Vieira, Sandra Magina, Dmitry V. Evtuguin, Ana Barros-Timmons

**Affiliations:** CICECO—Department of Chemistry, University of Aveiro, 3810-193 Aveiro, Portugal

**Keywords:** lignin, polyols, cyclic carbonates, polyurethanes, non-isocyanate polyurethanes, bio-based materials

## Abstract

Currently, the pulp and paper industry generates around 50–70 million tons of lignin annually, which is mainly burned for energy recovery. Lignin, being a natural aromatic polymer rich in functional hydroxyl groups, has been drawing the interest of academia and industry for its valorization, especially for the development of polymeric materials. Among the different types of polymers that can be derived from lignin, polyurethanes (PUs) are amid the most important ones, especially due to their wide range of applications. This review encompasses available technologies to isolate lignin from pulping processes, the main approaches to convert solid lignin into a liquid polyol to produce bio-based polyurethanes, the challenges involving its characterization, and the current technology assessment. Despite the fact that PUs derived from bio-based polyols, such as lignin, are important in contributing to the circular economy, the use of isocyanate is a major environmental hot spot. Therefore, the main strategies that have been used to replace isocyanates to produce non-isocyanate polyurethanes (NIPUs) derived from lignin are also discussed.

## 1. Introduction

**Figure 1:**
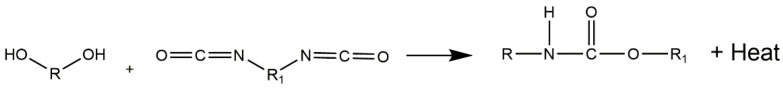
Schematic representation of the reaction between a polyol and a diisocyanate to form the urethane linkage.

Polyurethanes (PUs) are among the most multifunctional polymeric materials. They are traditionally produced by the polyaddition reaction between a polyol (R–OH) and a diisocyanate (R–NCO), affording polymers that contain urethane linkages (–NH–CO–O–), also called carbamate esters, as schematically shown in Figure 1.

The chemical structure of PUs contains soft segments and hard segments, the polyols being responsible for the soft part that contributes to the flexibility of PU chains, whereas isocyanates form hard segments and provide PU chains with rigidity. PUs have interesting properties such as low density, low thermal conductivity, mechanical strength, good chemical, and abrasion resistance [1,2]. These properties can be easily engineered by tuning their composition to suit various products such as foams (rigid and flexible), coatings, films, adhesives, elastomers, and other polymeric products that have versatile applications [3].

Industrially, almost all building blocks to produce PUs are derived from petroleum resources. In turn, isocyanates are produced by the reaction between amines and phosgene. Phosgene is a highly toxic gas produced from chlorine and carbon monoxide, which presents high risk to human health [4]. On the other hand, society’s concern about the environment is one of the main issues of the 21st century. Recently, the Paris agreement signed by 196 countries aims for the world to be climate-neutral until 2050, which means reducing greenhouse gas emissions by 80% compared to the level of 1990 [5]. Concurrently, the PUs market size was valued at USD 72.82 billion in 2021 and is expected to expand at a compound annual growth rate (CAGR) of 4.3% from 2022 to 2030 [6]. In this context, both industry and academia have been developing strategies to replace or decrease the use of petrochemical resources and hazardous products with renewable and safer products that have at least the same characteristics as those of conventional products. One of the building blocks of polyurethanes, polyols, is already being produced using renewable resources, especially from vegetable oil. The main players in this kind of market are BASF SE, Bayer, Dow Chemical, Huntsman, Covestro, and Cargill [7]. Likewise, other industries are exploring new businesses involving market niches for their byproducts, for example, the pulp and paper industry.

**Figure 2:**
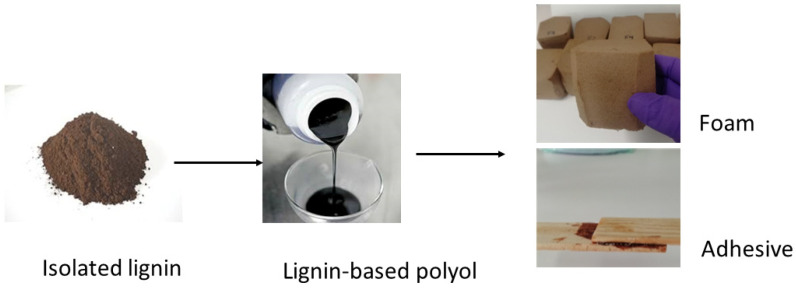
Polyurethane products from lignin-based polyol (LBP).

Presently, the main purpose of the pulp and paper industry using the kraft pulping process is to remove enough lignin to separate cellulosic fibers from one another to produce suitable pulp and paper. In turn, up to now, the lignin present in the black liquor has been used essentially for heat and power production after the recovery of chemical reagents [8]. However, lignin contains some special characteristics, such as the abundance of hydroxyl groups (OH) that can contribute to the development of a novel generation of bio-based polyols and PUs, adding value to the byproduct of the pulp industry whilst reinforcing the circular economy and biorefinery approach [9,10]. Figure 2 shows the main products involved in the transformation of lignin brown powder into a liquid polyol and finally into PUs foams and adhesives.

Lignin is the most abundant renewable aromatic polymer source obtained from biomass and has a potential opportunity to partially replace polyols from petrochemical origin used in the production of polyurethane products such as foams and adhesives. However, despite the fact that lignin has the key chemical features (rich in OH groups) to be used as a polyol as well as the ability to be incorporated directly into PUs formulations, the number and type of OH groups depend on a variety of factors, such as the process and the nature of the wood used to extract the lignin (see Table 1 and Table 2). Moreover, achieving the reaction between the OH groups, especially phenolic OH, with isocyanate groups, is a remarkable challenge. This is due to the steric constraints, recalcitrance, and the highly variable chemical structure of lignin. Hence, chemical modifications to convert it into liquid polyols are required to produce PUs, generally designated as liquefaction [11]. This is achieved by the functionalization of lignin OH groups, making them more accessible, which eventually affects their reactivity, as discussed later. Three main processes have been used to convert lignin into liquid polyols of polyether type: (1) oxyalkylation with alkylene oxides or cyclic carbonates [9,10,12]; (2) liquefaction with polyhydric alcohols [13,14]; and, more recently, (3) the combination of both [15,16]. Although these methods have been reviewed in the literature, for different types of biomass [17,18,19], there are several gaps regarding the production of lignin-based polyols. Moreover, despite the extensive studies to produce bio-based polyols, the use of toxic isocyanate remains an issue. Nevertheless, some contributions concerning its replacement by renewable bioresources to produce non-isocyanate polyurethanes have been published, and the numbers have grown in the last years, as will also be discussed later [20,21].

Due to its structural properties and specific chemical features, lignin may contribute to enhance the mechanical properties of PUs as well as their thermal stability. In fact, lignin can improve the flame resistance of these materials [22,23,24,25,26] by generating stable char during combustion [24,25,26]. Furthermore, the fire-retardant effect of lignin can be further improved by combining lignin with other flame-retardant additives, such as metallic hydroxides and phosphorus-based compounds [27], increasing the amount of char.

**Figure 3:**
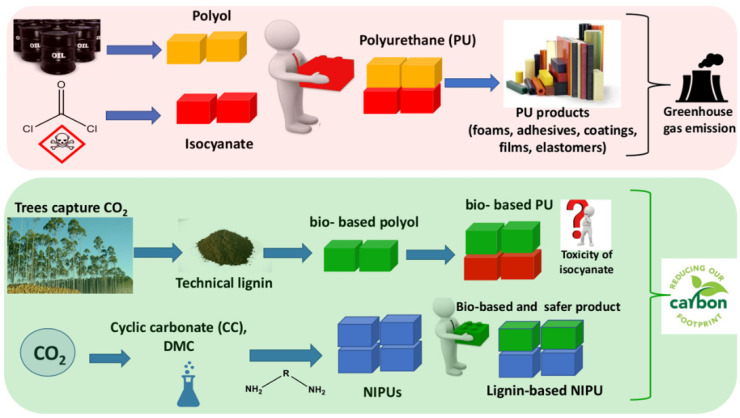
Lignin as a renewable building block to produce sustainable polyurethanes.

This review paper aims to place into perspective the main chemical strategies to use lignin as a building block to produce bio-based polyurethanes (Figure 3). This includes presenting and discussing the current state of technology to convert technical lignin into liquid polyols, taking into consideration the source of technical lignin, the quality of lignin, the processes variables of liquefaction methods, and the characteristics of lignin-based polyols produced, as well as the main constraints to convert lignin into liquid polyether-polyols and to characterize them. Additionally, the strategies that have been used to produce non-isocyanate polyurethanes (NIPUs) derived from lignin to yield bio and safer products will also be discussed.

## 2. Lignin and Its Structural Features

Lignin is one of the most abundant natural polymers present in lignocellulosic biomass, representing 20–30% of the biomass; hence, it is a potential sustainable raw material for the development of novel PUs [28,29]. However, the development of lignin-derived polymeric materials and subsequent applications requires knowledge about the raw material, especially because lignin is a complex polymer, and its isolation processes can influence the final product.

**Figure 4:**
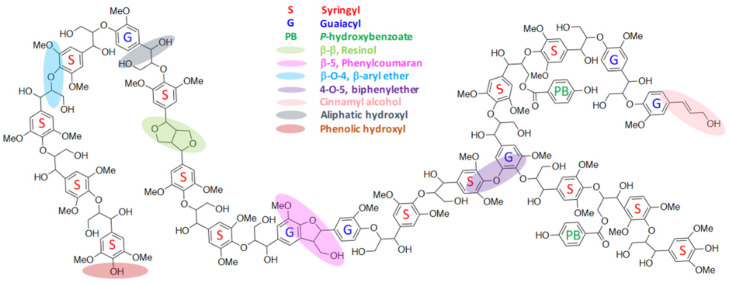
Example of hardwood lignin structure representation [30].

Native lignin has an irregular and complex structure with high molecular weight, and it is highly branched and amorphous [28]. Its chemical structure consists of different types of phenylpropane units (PPUs or C_9_), guaiacyl (G) and syringyl (S), and *p*-hydroxyphenyl (H) aromatic units, which differ in the number and position of the methoxyl groups on the aromatic ring, as presented in Figure 4 as an example of hardwood lignin structure. These phenolic substructures are linked by C–O bonds (ether bonds such as β–O–4, α–O–4, and 4–O–5) and C–C bonds, as well as other bonds such as 5–5, β–5, β–1, and β–β. The amount and types of these phenylpropane units of lignin depend on the plant species and environmental conditions. For this reason, the molecular structure of lignin cannot be given exactly by a structural formula, but estimated from the elements (C, O, H) and methoxyl group [30].

Hardwood trees (e.g., eucalyptus, beech) contain lignin with mainly S and G units. In turn, softwood trees (e.g., pine, spruce) contain lignins with mostly G units and low levels of H units. Grass lignin is made up of G, S, and H units in a wide range of proportions [31]. Furthermore, functional groups such as phenolic hydroxyl, aliphatic hydroxyl, carboxylic, and carbonyl groups attached to the basic phenylpropane skeleton of lignin have a great impact on their reactivity, optical properties, and dispersion characteristics. Methoxyl groups, though relatively less reactive, are also useful functional groups. From the amount of methoxyl group and elemental analysis (C, H, S, and O), the empirical formula of PPU (C_9_) can be deduced [32]. Table 1 summarizes the main structural features of lignin according to the species of trees.

**Table 1:** Structural features of native lignin [33,34,35,36,37].

Characteristics	Hardwoods	Softwoods
Total lignin, %	~18–26	~25–32
G units, %	~20–60	~95–98
S units, %	~40–8	0
H units, %	~4–10	~2–5
β–O–4 linkages, %	±50–65	~40–45
OH phenolic, per 100 C_9_	~10–20	~20–30
OH aliphatic, per 100 C_9_	~110–115	~115–120
OCH_3_, per 100 C_9_	~140–160	~90–95

## 3. Technical Lignins

Lignin can be isolated from different plants, but also from chemical pulping streams, which results in a technical lignin. The major production of industrial lignin comes from the pulp and paper industry, where most of the side streams are in the form of black liquor (annually, around 50–70 million tons are produced) which is mainly burned to supply internal energy and pulping reagents [38]. Commercially available technical lignins are often kraft, soda, and lignosulfonates associated with the corresponding industrial processes for wood delignification and cellulosic pulp production, e.g., kraft (using NaOH and Na_2_S), soda (using NaOH), and sulfite (using aqueous sulfur dioxide), respectively. Another technical lignin is organosolv lignin, obtained by the delignification of wood using a mixture of water and organic solvents (mainly ethanol) with or without catalysts. The native lignin structure changes minimally during the organosolv process and the organosolv lignin is a good option for further functionalization/modification. Yet, this process is not in industrial use, because of technical concerns, extensive corrosion of the equipment, high energy consumption due to the solvents recovery process, and the lower quality of the pulp produced when compared to the kraft process [39,40]. Amid industrial processes, the kraft process is the most widely used (more than 90% of mills). After the kraft pulping, the black liquor is obtained (14–18% of solids), which contains mainly lignin as well as other chemical species, such as carboxylic acids and inorganics salts [41]. The black liquor is usually concentrated to 70–80% in a series of evaporation effects and burned in a recovery boiler to recover inorganic reagents and to produce steam and electricity. However, in most cases, the amount of black liquor exceeds the design limits of the recovery boiler, which represents the “bottleneck” of the process. A convenient way that can minimize this “bottleneck” is the acidification of black liquor. This leads to the precipitation of lignin from the black liquor which can subsequently be used for higher-value-added applications.

Regarding the technologies available to separate the kraft lignin from black liquor by acidification, the techniques are based on changing the solubility of lignin or fractioning [41,42,43]. The MeadWestvaco Corporation Company was one of the first to carry out the precipitation of kraft lignin using sulfuric acid at pH 2–3; this technical lignin is marketed as Indulin™. Kraft lignin obtained by acidification using mineral acids has noticeable amounts of ash, sulfur, and sugars, and the yield can be affected by high filtration resistance [41]. To avoid these constraints, Inventia company and Chalmers Technical University jointly developed a lignin extraction process based on the precipitation with CO_2_ and acid, named the LignoBoost^®^ process (process owned by Metso corporation). First, the black liquor is acidified with CO_2_ at pH 9.0–10.5, precipitating the lignin. After flocculation, diluted sulfuric acid is added. In the last step, the lignin is isolated by filtration [43]. Presently, the kraft lignin isolated by the LignoBoost^®^ process is commercialized by the Domtar company and marketed as BioChoice lignin. Recently, Stora Enso company is also producing LignoBoost lignin at 50,000 tons per year [44].

**Figure 5:**
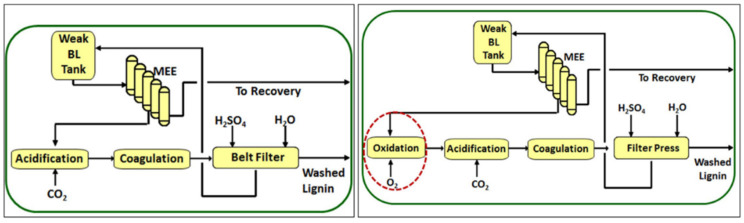
(**Left**) LignoBoost process; (**right**) LignoForce^TM^ [45].

Although the LignoBoost^®^ process provides lignin with high purity and solves the problem of filtration resistance that occurs with the acidification using strong acids, concerns about sulfur compounds (hydrogen sulfide, methyl mercaptan, dimethyl sulfide), that are malodorous and are hazardous to the health of humans, remain. FPInnovations group developed, in collaboration with NORAM Engineering company, the LignoForce^TM^ process [45]. In this approach, before the acidification of black liquor with CO_2,_ the liquor is oxidized with O_2_ until the sulfide concentration is reduced to a specific level and then follows the acidification and filtration steps, as illustrated in Figure 5 [45]. In 2015, the LignoForce^TM^ process was implemented by West Fraser company in Hinton pulp mill (Canada) with a capacity of extraction of lignin of around 30 tons per day [44].

The characteristics of technical lignin have great impact on its liquefaction process as well as the properties of the polymeric materials produced. These are determined by its purity, physical and chemical properties, and structural features which, in turn, are highly influenced by the pulping processes. In fact, most of these processes involve the cleavage of ester and ether linkages which can affect lignin reactivity for further chemical modifications [11]. Table 2 summarizes the basic characteristics of the main technical lignins.

**Table 2:** Characteristics of technical lignins.

Technical Lignins	Kraft	Lignosulfonate	Soda	Organosolv	References
Purity, %	88–95	50–70	88–95	97–98	[35,36,43]
Sulfur, %	1.0 to 3.0	3.5 to 8.0	0	0	[35,36,43]
Total OH groups, mmol/g lignin	5.0–6.0	4.0–4.5	4.0–6.0	5.0–10.0	[44,45,46,47]
MW, g/mol	1500–5000	1000–10,000	800–3000	500–5000	[35,43,46]
Tg, °C	140–150	130	140	90-110	[35,43,46]
Polydispersity	1.0–3.5	6–8	2.5–3.5	1.5–2.5	[35,43,46]
Trade names	Indulin, Curan, BioChoice	-	Sarkanda, BioLignin	Alcell	[7,39,43,48]
Product status	Industrial	Industrial	Industrial	Pilot scale	[36,43]

When technical lignins are used for the preparation of polymeric materials, especially as a polyol in PUs synthesis, the quantification of hydroxyl groups and determination of molecular weight of lignin are the main chemical features but can be a constraint. In fact, both of these characteristics are related to the reactivity of lignin towards isocyanates, final properties, and applicability of PUs [46]. The reactivity of hydroxyl groups is highly dependent on steric hindrance factors, which explains why aliphatic OH groups are more reactive than phenolic OH groups [47]. In addition, some technical lignins can present high molecular weight and a highly branched structure, which hinders their reactivity towards isocyanates [48]. Other issues related to the application of lignin in PUs materials are the dark color that can restrain some applications, such as coatings. In addition, kraft lignin and lignosulfonates have the problem of odor due to the presence of sulfur derivatives. Fortunately, the main issues can be solved by choosing the appropriate source of lignin and/or functionalizing the lignin via liquefaction processes.

With regard to the source of technical lignin, as already mentioned, the kraft lignin is the largest source of lignin, which has been isolated from black liquor using strong acid and CO_2_ (LignoBoost^®^ and LignoForce^TM^). Besides the influence of pulping processes, the isolation methodologies of lignin from black liquor can also affect its functionalization [37]. Indeed, kraft lignin obtained by acidification using mineral acids (e.g., Indulin lignin) has a slightly larger content of ash and sulfur than lignin obtained by LignoBoost and LignoForce^TM^, and may also present different structural features [49,50]. To understand the chemical structural differences between lignins obtained by the acidification and LignoBoost^®^ processes, Hu et al. [42] analyzed two commercial lignins, BioChoice and Indulin, obtained by the LignoBoost^®^ process and acidification, respectively. BioChoice and Indulin™ showed similar structures, but BioChoice showed a higher phenolic hydroxyl group content and lower methoxyl and β–O–4 contents. Hence, the researchers attributed this to the fact that the lignins were obtained using different pulping conditions.

LignoBoost™ and LignoForce^TM^ both allow obtaining lignin with high purity and similar chemical and structural characteristics, but the LignoForce^TM^ process minimizes, or can eliminate, the emission of TRS (total reduced sulfur) responsible for strong odor [45].

## 4. Synthesis of Lignin-Based Polyether Polyols

Polyols are nucleophilic compounds that contain two or more hydroxyl groups per molecule. They are classified according to their molecular weight as monomeric or polymeric polyol. Polymeric polyols are mainly used in the food industry (as sweeteners) and in the polymer industry. The latter is mainly interested in polymeric polyols such as polyether, polyester, polycarbonate, and acrylic polyol. Most commercial polyols are polyether and polyester polyols obtained from petrochemical sources. Since biomass residues such as vegetable oils (soybean, castor, sunflower, fatty acids, etc.), sugars, and lignin, among others, are rich in hydroxyl groups, the petrochemical sources can be partially replaced by these biomass residues, contributing to a more sustainable and resource-efficient development. In this context, both industry and academia have been making efforts to set up strategies to replace or reduce the use of the petrochemical resources, developing bio-based polyols from renewable resources that have at least the same characteristics as conventional products and are economically feasible.

Indeed, bio-based polyols derived mainly from sugars and vegetable oil are already commercialized. For example, the Dow company has been selling polyols derived from fatty acids, with the trade name RENUVA™, for use in the production of PUs adhesives and sealants. Another commercial bio-polyol is JEFFADD B650 ™ (Huntsman company) derived from soybean to produce rigid foams and coatings [51]. Among the various types of biomass, lignin is the most abundant, especially kraft lignin, for which production increased by 150% from 2014 to 2018 [52] with the advantage that, compared to vegetable oils, lignin does not compete with the food supply. At the moment, lignin-based polyols (LBPs) are not industrially available, but Dessbesell et al. [52] estimated that this product has around 3000 kt potential production according to the market polyol value and size. Furthermore, a technical economic assessment carried out by this research group showed that the lignin-based polyol is a feasible bio-substitute of petroleum-based polyols with a minimum selling price of USD 1623/ton [53]. In the following subsections, the main chemical modifications of lignin to obtain polyether polyols, the main characteristics of polyols from lignin, and the challenges involved to produce and characterize them will be discussed.

### 4.1. Lignin-Based Polyol via Oxyalkylation Reaction

**Figure 6:**
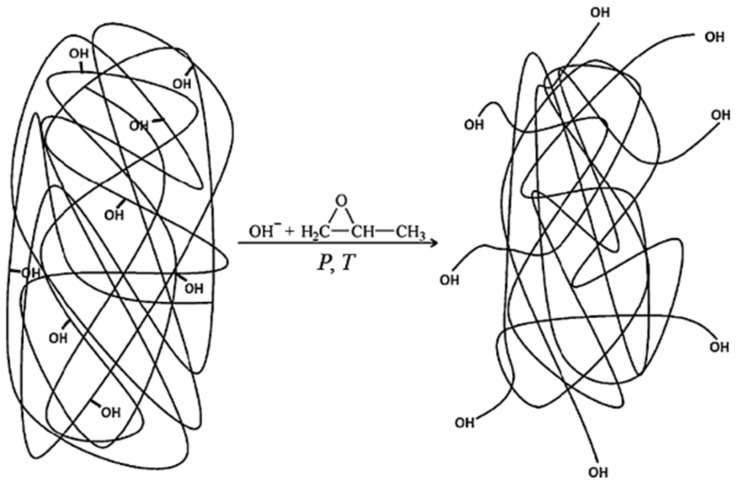
Scheme of oxypropylation reaction of biomass with PO [18].

Further to the review work of Alinejad et al. [46] regarding a variety of strategies to chemically modify lignin to obtain lignin-based polyether polyols, in this subsection, a more detailed discussion is given regarding those that have been more frequently reported in the literature. For example, the oxyalkylation of lignin can be carried out using alkylene oxides, such as ethylene (EO) or propylene oxide (PO), by ring-opening polymerization yielding grafts of poly(propylene oxide or ethylene oxide) generally under basic conditions, high pressure, and high temperatures (usually 150–170 °C and 10–20 bar), as schematically illustrated in Figure 6 [18]. Due to the safety risks involved in the manipulation of EO, in this review, focus will be given to examples involving the use of PO, which is still a rather dangerous chemical to handle but does not require as strict safety operation conditions as EO. Acid catalysts can also be used, although the reaction is less efficient with phenolic OH groups [54]. In the oxyalkylation reactions, the hydroxyl groups of lignin, particularly the phenolic ones that are inside the molecule and difficult to access, are liberated from steric and/or electronic constraints, and, at the same time, the solid lignin becomes a liquid polyol, because of the introduction of multiple ether moieties.

It is worth mentioning that oxyalkylation reactions do not increase the number of hydroxyl groups; instead, they increase their availability and eventually their reactivity [55,56]. Cateto et al. [9] converted different lignins into liquid polyols by a chain extension reaction with PO using KOH as a catalyst. The characteristics of the LBP proved to be in the range of those of typical commercial polyols employed in the production of polyurethane rigid foams.

Glasser and his coworkers modified lignins using different conditions at high temperatures and pressures, paving the way for further investigations on them in recent years [57]. Lignin-based polyurethanes were also prepared using lignosulfonate via oxypropylation, and the ensuing products showed good mechanical properties [58]. Likewise, wheat straw soda lignin was converted into polyols for further polyurethane production using PO [54]. Indeed, many studies applied oxypropylation using PO to expose phenolic OH groups of lignin, making them more reactive by increasing their accessibility and affording aliphatic OH groups. Nevertheless, PO involves considerable risks due to its high vapor pressure, flammability, and toxicity, and still it is necessary to use homologated equipment to ensure safety conditions against explosions caused by sudden uncontrolled exothermic polymerizations [12]. Furthermore, the reactions lead to the grafting of oligomeric chains and the simultaneous formation of homopolymer, reducing the yields of oxypropylation. The homopolymer can be removed by solvent extraction with *n*-hexane [9,10,48,57] or cyclohexane [59]. However, the solvents are toxic and flammable as well. To overcome this problem, more recently, the use of cyclic carbonates has been studied as an alternative to PO, because this class of compounds appears to be more compatible with the green chemistry requirements.

Cyclic carbonates have been used over the years as reactive intermediates and inert solvents in several applications, such as carrier solvents applied in cosmetics and medications, as well as alkylating agents of phenolic resins. Cyclic carbonates can react with amines, alcohols, and carboxylic acids to initiate ring-opening polymerizations [60]. The first oxyalkylation of lignin using cyclic carbonates was reported in 1953 and patented by Monson and Dickson [61]. This patent described the oxyalkylation of a lignosulfonate using various types of cyclic organic carbonates, but this synthetic route has only recently been applied to other biomass residues. Oxyalkylation of lignins and tannins was recently performed using propylene carbonate (PC), yielding products that are structurally similar to those obtained by oxypropylation with PO [12,59]. Although PO is less expensive than cyclic carbonates, the latter present advantages compared to PO. Namely, the oxyalkylation with cyclic carbonates does not require high-pressure equipment nor the use of additional solvent because these compounds are high-boiling-point liquids and can act as reagent and solvent. In fact, their action as a solvent is an advantage in the alkylation of aromatic substances that have high melting points [60].

There are several cyclic carbonates commercially available in the market, including propylene carbonate (PC), ethylene carbonate (EC, glycerol carbonate (GC), and vinyl ethylene carbonate (VEC). Yet, PC and EC are the most commonly used as alkylating agents of phenols [60] and more recently in the oxypropylation of biomass [12,59,62]. This may be associated with the fact that, for instance, PC presents interesting features, such as low toxicity, high boiling point, low vapor pressure, and low inflammability (Table 3), making it an interesting solvent and reagent that can be a substitute of PO for the oxypropylation of lignins.

**Figure 7:**
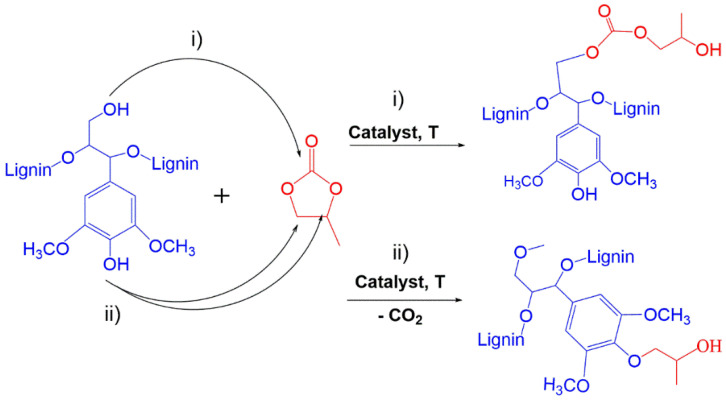
Scheme of oxypropylation of lignin with PC [18].

The mechanisms involved in the reaction of cyclic carbonates with lignins, considering lignins as a polyphenol, are similar to the ring-opening polymerization reactions of oxiranes with lignins (e.g., PO); thus, the structures obtained are similar. Depending on the type of hydroxyl group involved and the reaction conditions, distinct reaction paths can be involved. For example, in the case of PC and lignin (Figure 7), the reaction proceeds in two steps: (1) the OH groups of lignin are activated by basic catalysts such as K_2_CO_3_, KOH, or organic bases; (2) the oxyalkylation reaction occurs via opening the cyclic carbonate ring by the attack of phenoxide to the alkylene carbon of the cyclic carbonate, leading to chain extension. This second step can involve the formation of either ether linkages, as in the case of PO, or ester linkages. Cyclic carbonates react with aliphatic hydroxide groups of lignin by transesterification, leading to chain extension via the formation of carbonate linkages [12,64] (path (i) in Figure 7). Concomitantly, the reaction of the phenoxide of lignin with cyclic carbonates will afford ether linkages and elimination of carbon dioxide, as illustrated in path (ii) in Figure 7. Carboxylate groups of lignin can also react via alkylation, yielding ester linkages [59,65]. The reactions with phenoxide and carboxylate groups are always accompanied by the elimination of carbon dioxide [66].

The characteristics of LBP (e.g., OH content, viscosity, degree of substitution, and degree of polymerization) obtained by oxyalkylation with PO can be controlled or improved by adjusting the parameters of oxyalkylation reactions, such as catalyst, time, temperature, and the ratio molar of solvent/lignin, and this technology is already well studied [18,55]. On the other hand, studies on the influence of the reaction parameters of oxyalkylation of lignin using cyclic carbonates on the final product are still little exploited. For oxyalkylation of alcohols, amines, and thiols, temperatures from 100–200 °C have been reported, whilst the temperatures required for the reaction of cyclic carbonates with alcohols are in the range of 150–200 °C [60]. Notice should be taken that attention is required when the reaction is carried out with lignin, as high temperatures in the alkaline medium may lead to structural changes in lignin [67]. Some studies have investigated the ring open polymerization of cyclic carbonates (especially propylene carbonate) using different raw materials as initiators and different catalysts [62,63,65]. For example, Kühnel et al. [63] studied the influence of reaction parameters on the ensuing bio-based polyols in order to obtain information to control and optimize the oxyalkylation of organosolv lignin with PC. For that purpose, the influence of catalyst, catalyst/lignin ratio, reaction time, temperature, and the amount of PC were investigated. Specific attention was given to examining the effect of temperature reaction (100–170 °C) on the addition of propyl units and the degree of substitution (DS). It was observed that the DS and the number of propyl units increased with the increase of temperature, which demonstrated that the density of oxypropylated lignin is highly dependent on temperature, the highest DS and the grafted chain length being achieved at 170 °C. The same trend was observed by Duval et al. [68] using soda lignin and VEC to produce biobased polyol, who reported that the best conversion of phenolic OH groups was achieved at 150 °C.

Since the reaction of OH groups of the lignin with terminal carbonate structures requires high temperatures, other side reactions can also take place. To avoid these side reactions and improve the conversion of phenolic OH, as well as reduce the time of reaction, catalysts are used. Yet, in the reactions involving phenolic groups as initiators, only basic catalysts yield chain extension. The most used basic catalyst is K_2_CO_3_, but organic bases such as 1,8-diazabicyclo [5.4.0] undec-7-ene (DBU) have also been mentioned as suitable catalysts for oxyalkylation reaction [69,70]. For instance, several classes of amidines and guanidine have been reported as good nucleophilic catalysts activating the precursor in ring-opening reactions [71,72]. Kühnel et al. [63] and Vieira et al. [64] used different organic and inorganic catalysts to investigate the influence of the catalysts on oxyalkylation reactions. Both studies indicated that DBU allowed attaching more oxypropyl units to lignin when compared to inorganic bases such as K_2_CO_3_. Duval et al. [68] reported attempts using another strong organic base, 1,5,7-triazabicyclo [4.4.0]dec-5-ene (TBD) as a catalyst in the reaction of the lignin with VEC, to reduce the time of reaction and to avoid the formation of high-molecular-weight byproducts when long times of reaction were used. Contrary to what was expected, the conversion of phenolic OH groups was not accelerated, and even seemed to favor crosslinking reactions. In fact, the chemical nature of the carbonate, namely, the presence of the vinyl group, must be kept in mind. Despite the divergences of some studies on the influence of organic base catalysts on oxyalkylation reactions, these compounds are efficient as a catalyst to produce bio-based polyols.

Another critical parameter of this type of reactions is the solid/liquid ratio that is related to the amount of cyclic carbonate, as during the reaction the cyclic carbonate acts both as reagent and solvent. Therefore, cyclic carbonates are used in excess to completely dissolve the lignin. Recent studies have indicated that amounts of cyclic carbonates, especially PC, range between 10–30 equivalent molar, and its increase results in a slight increase of the grafting of chains from lignins [63]. In addition, related to the use of excess of cyclic carbonates, Duval et al. [68] made several attempts to recover the excess of VEC at the end of the reaction via the precipitation of the modified lignin in toluene. However, in some cases of preparation of PUs, cyclic carbonate recovery is not required [73].

A low amount of cyclic carbonate can be used in the oxyalkylation of lignin, but the mixture results in a slurry that is difficult to stir. Recently, Zhang et al. [15] performed the oxyalkylation of kraft lignin with EC using polyethylene glycol as cosolvent to circumvent the issue of viscosity to produce suitable rigid foams. Along the same line, the Hexion Inc. company recently disclosed the process to produce PUs by oxyalkylation of lignin using cyclic carbonates and polyhydric alcohols [16].

It is worth mentioning that the oxypropylation of biomass is also associated with the occurrence of side reactions, namely, the formation of significant amounts of homopolymer (e.g., poly(propylene) glycol), which has to be removed by solvent extraction with *n*-hexane [55,59]. Although the homopolymer is able to react with isocyanate to produce PUs, its proportion can affect the polyol quality and the yield of oxyalkylation reaction. The formation of homopolymer alongside oxyalkylation of biomass using cyclic carbonates has been mentioned in some works [62,64,74], and even crosslinking reactions have been reported involving chain coupling through transesterification reactions of biomass with cyclic carbonate [65], but this issue has not been discussed in depth. Usually, after the oxyalkylation, the product obtained is a mixture of oxyalkylated biomass, some unreacted cyclic carbonate, and perhaps homopolymer. Recently, Vieira et al. [64] performed the oxyalkylation of LignoBoost^®^ kraft lignin with PC at 170 °C during 2.5 h using different catalysts and investigated the formation of homopolymer by high-performance liquid chromatography. It was concluded that only a minor proportion of PC (3–15%) was converted to propylene glycol/homoligomer.

Table 4 summarizes the main parameters evaluated regarding the oxyalkylation reaction of lignin using alkylene oxides (PO) and cyclic carbonates, and the characteristics of the ensuing LBP.

### 4.2. Lignin-Based Polyol via Liquefaction with a Polyhydric Alcohol

An alternative to oxyalkylation with PO or cyclic carbonates is the liquefaction of lignin using polyhydric alcohols such as polyethylene glycol (PEG), polypropylene glycol (PPG), ethylene glycol (EG), glycerol, or a combination of them in the presence of acid or basic catalysts. The most frequently used conditions include the use of sulfuric acid (H_2_SO_4_) and moderate–high temperatures (110–180 °C) under atmospheric pressure [13,17,76,77,78]. When the reaction is carried out using base catalysts, higher temperatures are required; in general, 250 °C [14].

**Figure 8:**
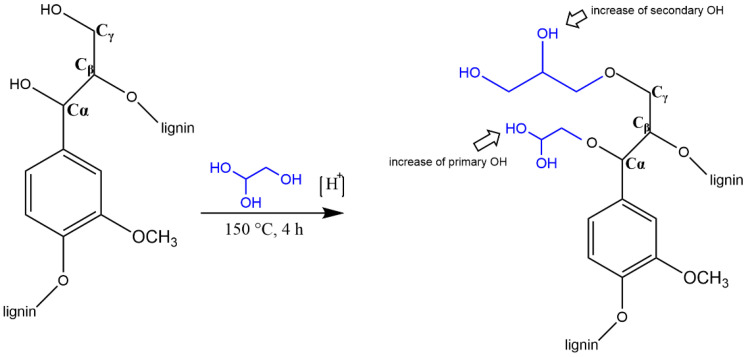
Proposed reaction scheme of lignin with glycerol during wood liquefaction using PTSA (ρ-toluene sulfonic acid) as a catalyst based on carbon assignments, adapted from [81].

During the acid liquefaction of lignin, degradation and repolymerization are the main processes that occur. The hydroxyl groups of lignin are linked to the PEG and or glycerol via ether bonds in the liquefied product, but the mechanisms involved in this process are quite complex, and recondensation reactions have also been proposed [79]. The occurrence of these recondensation reactions between lignin and solvents takes place at the same time and competes against the liquefaction reaction. If these reactions are dominant, the percentage of residue increases, leading to lower efficiency of the liquefaction [80]. Many possible reaction paths have been proposed to explain the occurrence of side reactions and depolymerization of lignin. Jasiukaityté et al. [79] studied the structural changes of lignin during the liquefaction with glycerol and ethylene glycol. They confirmed the grafting of glycerol and diethylene glycol onto lignin at the Cα and Cγ positions associated with the increase in primary and secondary hydroxyl group content using ^31^P NMR spectroscopy (Figure 8). The liquefaction of lignin was predominantly due to the condensation reactions between the aromatic lignin sub-units, as indicated by the decrease in total phenolic hydroxyl group content, followed by the incorporation of glycerol and diethylene glycol moieties [79,81].

Typically, the product obtained from the liquefaction process is a mixture of liquefied lignin and solid residue, the latter being a limiting factor of the liquefaction yield. To overcome or avoid the formation of residue and recondensation reactions, reaction parameters such as temperature, time, catalyst loading, and lignin/solvent ratio can be optimized. Furthermore, the choice of solvent type also has an important role in the production and characteristics of the polyol since the polyhydric alcohol works as solvent and polyol [17]. For instance, a mixture of polyethylene glycol (PEG 400, Mw: 400 g/mol) and glycerol is the most commonly used as liquefaction solvent to produce polyols for PUs foam production [13,76,78,82]. In addition, the use of glycerol has been reported to prevent recondensation reactions [83]. Regarding the solvent: lignin ratio (*w*/*w*), significant amounts of solvent are necessary to liquefy the lignin, which ranges between 3:1 and 9:1, to obtain high efficiency and minimize recondensation reactions. In fact, most of the studies found that the optimal solvent/lignin ratio is 5:1.

In acid liquefaction, the presence of a catalyst is very important to improve the liquefaction extension, to reduce the temperature and time of reaction. As mentioned earlier, the most used catalyst is H_2_SO_4_, and the effect of different loadings of this catalyst has been investigated in several studies [17,19,84]. Most of the studies involving the acid liquefaction of lignin achieved high efficiency of conversion into polyol using temperatures in the 130–180 °C range and 1 h of reaction. Generally, the H_2_SO_4_ catalyst loading lies within the 1–6% (*w*/*w*%) interval [13,19,82,85,86]. However, the dosage should not be too high (over 4%), to avoid the recondensation reaction [13,84]. Table 5 summarizes the main parameters of acid liquefaction of lignin using H_2_SO_4_ and their impact on the formation of solid residue discussed in the literature.

Recently, Silva et al. [14] evaluated the use of organic acids (lactic, acetic, and citric acid) as catalysts in the liquefaction of kraft lignin using polyhydric alcohol. These catalysts have great potential to replace the conventional inorganic acids since they can be obtained from renewable resources and turn the process more sustainable. Interestingly, lignin has also been successfully liquefied using the microwave heating method, which allows the reduction of reaction time and may reduce the cost of the process [83,87,88,89]. Despite the fact that the acid liquefaction can be an option to liquefy lignin, the implementation of this approach remains a challenge due to the occurrence of side reactions and the formation of considerable solid residue.

### 4.3. Quality of Lignin-Based Polyol and Its Characterization

**Figure 9:**
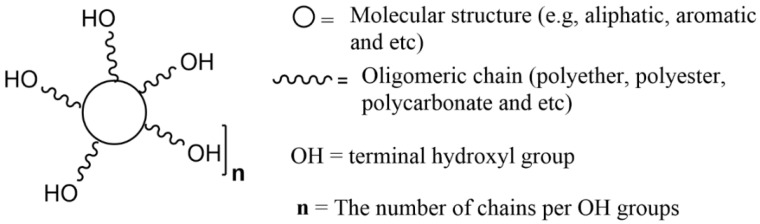
Scheme of the general formula of polyol, adapted from [2].

For petroleum-derived polyols used in the preparation of polyurethanes, the main characteristics that are normally quantified are the content of hydroxyl groups (hydroxyl number, I_OH_), the acid number of polyol, water content, viscosity, and molecular weight as they have a direct impact on the formulations. The required values depend on the type of PU to be produced, i.e., foams, films, or adhesives [3]. Figure 9 schematically illustrates these main characteristics.

The hydroxyl number (I_OH_) represents the amount of hydroxyl groups available for the reaction with isocyanates. The number can be determined by the reaction of the terminal hydroxyl groups using organic anhydrides (acetic anhydride or phthalic anhydride) [3]. The range of I_OH_ values of polyols required for the production of PUs is quite wide, e.g., polyether polyols used to produce rigid foams have I_OH_ values between 300–800 mg KOH/g, while for PU adhesives production the range of I_OH_ values is usually lower [3,90].

The acid number is related to the amount (in mg) of potassium hydroxide needed to neutralize one gram of sample [91]. The importance of the acid number is due to the fact that the presence of residual acidity decreases the catalytic activity of the tertiary amines used in the reaction of polyols with isocyanates to produce PUs. For polyurethane formulations, the acid number should be around 0.05–0.1 mg KOH/g for polyether polyols, and 2 mg KOH/g for polyester polyols [18].

Water content is also an important issue since isocyanate groups are highly reactive towards water; thus, it competes with the hydroxyl groups of polyols during the synthesis of PUs. The determination of this characteristic is carried out by the classical Karl Fischer method, and the required values for producing PUs are between 0.05–0.1% [3].

Viscosity indicates the processability of a polyol, which can be determined using a Brookfield viscosimeter or a rheometer. Furthermore, it can provide an indication of the reactivity of a polyol with an isocyanate to afford the final PU. It was observed that polyols with low reactivity (0% primary hydroxyl, i.e., having only secondary hydroxyl groups) have the lowest viscosity increase over time. In turn, highly reactive polyols, having 85–100% primary hydroxyl content, have the highest viscosity increase over time [3]. For the production of PUs, the range of viscosities can be wide, depending of the type of PU, but must be below 300 Pa.s [18].

The molecular weight (MW) and polydispersity of polyols can be rigorously determined by size exclusion chromatography (SEC), also known as gel permeation chromatography (GPC). The molar mass sensitive detection is based on both viscometry and universal calibration [92]. The MW is an important parameter because this feature will impact the network structure and crosslinking density, which influences the physical properties of PUs. Generally, for use in the production of polyurethane foam (PUF), polyols that have large MW will lead to amorphous regions that impart flexibility to Pus, whilst polyols with low MW will lead to rigid PUs [3]. Regarding the required values for PU production, in general, polyols have MW in the range of 400–5000 Da, depending on the type of PU product [93].

Since lignin has a very complex structure and variable characteristics, LBPs must be well characterized in order to better predict the behavior of the ensuing PUs and understand the relationship between the structure of the polyol and the properties of PUs. The difficulty in characterizing these structures has led many researchers to characterize only the most essential features of LBPs such as the I_OH_, viscosity, and MW, as is normally performed for petroleum-based polyols, as mentioned earlier. Nevertheless, the characterization of LBPs can be quite complex and can present some challenges. Hence, it must be performed carefully, and sometimes different methods need to be used when compared to their petroleum-based counterparts. For example, the determination of the number of terminal hydroxyl groups of petroleum-based polyols involves the esterification of the alcohols with acetic anhydride or phthalic anhydride using solvents/catalyst such as pyridine or N-methylimidazole [94]. However, the dark color of LBPs can induce significant errors during the conventional titration using phenolphthalein as an indicator. Potentiometric titration circumvents the problem, but sterically hindered alcohols and phenolic alcohols present in LBPs can result in some inaccuracies. The alternative is to use ^1^H NMR and ^13^C NMR spectroscopies to quantify the number of hydroxyl groups [95]. Complimentary to this, Granata and Argyropoulos [96] developed a methodology that allows differentiating the hydroxyl groups by ^31^P NMR. However, for the use of these methods, internal standards are required, but the NMR signals of the internal standard can either overlap with signals from the lignin, or have poor stability due to degradation of the internal standard. In addition, phosphorus-containing lignin derivatives have limited stability in solutions, and ^31^P NMR analysis should be carried out immediately after derivatization. To overcome this drawback, the application of the PULCON (pulse length-based concentration determination) method can be useful to determine the hydroxyl groups of lignin [97]. All these results are normally reported based on the phenylpropane units (PPUs) [95]. Furthermore, the number of PO or cyclic carbonate units grafted onto lignin by oxyalkylation can be expressed as the degree of substitution (DS), and the number of oxypropyl units attached to each PPU as the degree of polymerization (DP), based on the ^13^C data and ^1^H NMR spectroscopy [34,75].

GPC is one of the most commonly used techniques to determine the average molecular weight of polyols. The eluents used can be organic solvents (THF, DMF, or DMSO) [98]. Polystyrene standards are frequently used to obtain the calibration curve, though in the case of lignosulfonates, polystyrene sulfonates standards have also been used. With regard to the detectors and characteristics of the columns, the ultraviolet (UV) or refractive index (RI) detectors are the most common, whilst the type of column depends on the molar mass range of the polymer and eluent compatibility. Yet, care should be taken for lignin-derived polyols because the UV detector is not always accurate, due to the different extinction coefficient of lignin fractions. Typically, mixed pore columns are preferred due to the larger range of linear calibration. However, LBPs can present some issues due to their complex structure. Indeed, not all lignins present good solubility in the eluents, and the calibration curve obtained using common polymer standards also may not be ideal, which introduces errors in the molecular weight measurement of LBPs [19,99]. Ideally, the use of previously characterized lignin samples with well-known molecular weight as calibration standards seems to be more attractive [64], as well as the use of light scattering detectors for SEC. Furthermore, multi-angle light scattering (MALS, typical angles 30–150°) combined with SEC equipment has been used to determine the absolute molecular weight of biopolymers, and is also a promising technique to determine the molecular weight of LBPs. The technique is based on the intensity of the light scattered by the sample, which is directly proportional to the molecular weight [98,100].

Additionally, the characterization of bio-based polyols, in particular, lignin-derived polyols, requires complementary characterization steps when compared to petroleum-based polyols. In fact, besides the determination of the main key features such as I_OH_ number, MW, viscosity, and functionality, other analyses must be included that depend on the liquefaction method used. For example, the amount of solid residue formed is a crucial feature when the LBP is produced via liquefaction using a polyhydric alcohol. Usually, the determination of residue formed is performed by extraction with solvents (e.g., acetone, dioxane), and after filtration, the solid remaining is obtained by weight difference. Combined with the determination of the amount of residue formed, further chemical and structural analyses of the residue provide information about the efficiency of the process, as well as the occurrence of condensation reactions [101]. On the other hand, polyols produced via oxyalkylation using alkylene oxides generally contain a significant amount of homopolymer which results from the reaction of the monomer with water and needs to be removed by extraction using *n*-hexane. The homopolymer content is determined by the gravimetric method after evaporation of the *n*-hexane [10]. In oxyalkylation with cyclic carbonates, the determination of the amount of homopolymer is achieved by precipitation of the lignin-polyol using diluted acid as hydrochloric acid (HCl), whilst the supernatant containing the homopolymer can be later characterized by high-performance liquid chromatography (HPLC) [64].

Souza et al. [19] published a review focused on the characterization of bio-based polyols produced via oxyalkylation and acid liquefaction, and presented the main challenges for industrial applications. According to the authors, the functionality of bio-based polyol and the high variability of the nature of the biomass are, in fact, the main challenges. While the functionality of conventional polyols is well known and fixed, the variability of the molecular structure of bio-based polyol makes it impossible to determine an exact value of functionality; instead, a range of functionality is determined. Another challenge is the high variability of lignin, which, as mentioned earlier, depends on its natural source and the extraction process used, both of which have a major impact on the final properties of LBPs, making it difficult to produce uniform polyols.

## 5. Lignin as a Building Block to Synthesize Polyurethanes

The use of lignin in the production of PUs can be carried out in different ways: unmodified, being directly incorporated into polyol formulations, after fractionation, or after chemical modification (in order to make it more reactive), alone, or in combination with other polyols [46,47,102,103,104,105,106,107]. Depending on its interaction with the isocyanate, lignin can act as filler or as reagent, i.e., as polyol, also referred to as cross-linker. Although the use of lignin without any treatment is widely reported, if its OH groups do not react with the isocyanate and become chemically bonded to the PU network, it should not be described as a building block. The direct exploitation of lignin, as a polyol on its own or blended with industrial polyols, is energetically and environmentally advantageous [108], and the ensuing biomass-based PUs are more biodegradable than those derived from petroleum-based polyols. Even though the direct use of lignin as the only polyol can be very appealing, generally, lignin macromonomers have low reactivity towards isocyanate groups yielding products without the desirable performance, and end up acting essentially as fillers [46,109,110]. An alternative to the chemical modifications discussed in Section 4 or fractionation (discussed next) is to blend it with petrochemical polyols. However, this can also be quite a challenge due to the highly polar nature of lignin in contrast with the relatively low polarity of polyether polyols [111]. Consequently, the lack of homogeneity of the polyol blend [111] is detrimental to achieving PUs with consistent mechanical properties. To minimize phase segregation associated with the poor miscibility of unmodified lignin in conventional petroleum-based polyols, the content of the unmodified lignin, as a partial replacement of petroleum-based polyol, in the ensuing PU is generally below 30 wt.% in order to reach an acceptable performance [105,107]. Notice should be taken that, unless otherwise specified, the values regarding the percentage of incorporation of modified or unmodified lignin refer to weight percent relative to the conventional polyol.

Recent advances to circumvent the drawbacks of direct incorporation of lignin as polyol in the production of PUs include (1) the use of diols and glycerol as compatibilizer and cross-linker, and (2) lowering the lignin molecular weight using solvent fractionation. Since kraft lignin is the most commonly produced technical lignin, most of the studies are focused on this type of lignin. For example, Haridevan et al. [112] recently evaluated the dispersion and solubility of kraft lignin in different types of polyols at room temperature for the production of polyurethanes based on microscopic, gravimetric, and rheological analyses. This study demonstrated that kraft lignin has different degrees of dispersion in various polyols, depending on the structural characteristics such as solubility parameter, molecular weight, and monomeric unit type. In fact, it was observed that a higher degree of dispersion of kraft lignin was achieved in the lower-molecular-weight diethylene glycol (106.1 g/mol) than in polyethylene glycol (400 g/mol). Although the low dispersibility of lignin in polyols has not yet been solved, systematic studies such as this one are important contributions to increase lignin dispersion. In addition, the performance of PUs can be improved by heating the polyol/lignin dispersions at 120 °C prior to the reaction, which enhances the disaggregation of lignin microparticles, yielding a better lignin dispersion within the polyol system [113,114]. On the other hand, reducing the kraft lignin molecular weight by solvent fractionation can enhance its miscibility and dispersion in the PU matrix, and consequently can improve some properties of the resulting PU such as the mechanical properties [115]. The use of binary organic solvent mixtures such as acetone–methanol [116], aqueous two-phase systems (ATPSs) composed of (NH_4_)_2_SO_4_ and ethanol [117], and the use of ionic liquids [118] are examples of strategies used to reduce the molecular weight of lignin as well as its heterogeneity. However, it is important to take into account that commercializing low-molecular-weight lignin has been economically unfeasible until now. Jiang et al. [119] performed a technical economic assessment of the fractionation of kraft lignin using organic solvents, where the minimum production selling price for the low molecular weight lignin was estimated to be between USD 700 and 1000, while the minimum selling price for the kraft lignin is USD 250/ton. Moreover, the environmental impact of such strategies needs to be taken into account.

As mentioned earlier, the use of unmodified or modified lignin as polyol in the production of polyols has several advantages. However, for some applications, the stiff character of the lignin needs to be compensated using other polyols as soft segments. Examples of this strategy include the use of polyethylene glycol (PEG) and polypropylene glycol (PPG), bio-based polyols such as castor oil [83,120,121,122], crude glycerol [120], and poly(ε-caprolactone) (PCL) [123], which counterbalance the stiffness of the lignin macromolecules yielding grafted and cross-linked PUs, offering the possibility of controlling the flexibility and/or rigidity [83,104,124,125]. Pan et al. [126] replaced the polyol by organosolv and kraft lignin on the production of rigid PUF, and the foams showed satisfactory structure and yield strength up to 30% of substitution of conventional polyol by lignin. Above this content, the viscosity increased considerably, which affected the uniformity of The cells. Unmodified lignin has also been used to produce flexible polyurethane foams, despite the intrinsic stiffness of lignin which favors the production of rigid foam. Yet, depending on the balance of the formulation, flexible foams can be obtained which are characterized by mostly open-cell structures [105]. Recently, Gondaliya and Nejad [107] replaced 20 wt.% of conventional polyols by organosolv and kraft lignin in the formulation of flexible polyurethane foams, which increased tensile, compression, tear propagation strengths, and thermal stability.

In addition to PUF, different types of lignins and LBPs have also been used to prepare other forms of Pus, such as adhesives [121,127,128], elastomers [129,130,131], coatings [132,133], and films [125]. Elastomeric PUs are a class of PU materials that have the characteristics of rubber, and their application has increased in recent years due to the high demand for advanced applications such as in biomedicine, shape memory materials, self-healing materials, and gel materials. Mechanical properties such as toughness, tensile strength, and high elongation at break are highly desirable for the production of PU elastomers, and the use of modified or unmodified lignin in the synthesis of PU elastomers has shown that it plays an important role in improving these properties, where a percentage of lignin up to 40 wt.% (based on mass of polyol) did not jeopardize their mechanical properties [134,135,136]. In addition, since lignin is a natural UV-blocker, due to the presence of chromophores, sustainable PU elastomers with UV resistance can be synthesized to be used as additives to packaging, textiles, automotive trims, etc. Recently, Li et al. [131] produced a UV-resistant transparent lignin-based PU elastomer up to 2 wt.% of lignin (relative to PU) with good tensile strength and high elongation at break, which was fixed onto the cotton fabric by hot pressing to yield lignin-based PU hot-pressed fabrics with excellent UV resistance, i.e., with an average value greater than ultraviolet protection factor (UPF) 50.

Another application of PUs is as coatings which consist of a polyurethane layer applied on the surface of a substrate for the purpose of protecting it. These coatings are widely used in military, automotive, packing, and aerospace industries. Besides the UV-resistance conferred by lignin, another intrinsic characteristic of this material is its antimicrobial activity. In this sense, lignin has also been explored in the production of PU coatings, showing good antimicrobial activity and UV resistance. Notice should be taken that most of these studies used less than 50 wt.% of lignin to avoid the brittleness of coating [137,138,139].

In turn, Llovera and coworkers [125] produced PU films using an unmodified organosolv lignin and PEG-600 as polyols and toluene diisocyanate (TDI) as isocyanate. After optimization of the synthesis parameters, namely, the molecular weight of PEG and the NCO/OH ratio, the authors substituted up to 70 wt.% of the PEG with lignin. The ensuing PU films exhibited satisfactory mechanical properties and improved thermal stability, where the onset decomposition temperature of the lignin PU films reached an average limit of 310 °C, regardless of the lignin content, and 260 °C for the PU film without lignin [125]. Furthermore, due to lignin’s inherent hydrophobicity, lignin nanoparticles (LNP) were added to PU emulsion to prepare composite films [140]. As a result, the water contact angle increased from 65.1° (for pure PU film) to 114° (for the PU film with 5 wt.% LNP). Avelino and coworkers [141] prepared Pus through a three-component system formed by coconut shell ethanosolv lignin, PEG-400, and TDI, using a solvent-free polymerization route. The authors assessed the effect of lignin content and NCO/OH ratio used in the formulations in the range from 0 to 50 wt.% and 1.0–1.5, respectively, on porosity and crosslinking degree of PUs. The ensuing lignin-based PUs displayed higher thermal and thermo-oxidative stability compared to the PUs without lignin, demonstrating the possibility of producing a variety of polymers for different applications. Besides the effect on the mechanical performance of lignin-derived PUs, in general, most studies reporting the direct incorporation of lignin in the polymer matrix resulted in the improvement of some properties or even in new ones, such as flame retardancy [26,27,142], ultraviolet (UV) blocking [131,143], and thermal stability [107,121]. The examples discussed so far prove that the presence of lignin has a crucial impact on the performance of the ensuing PU materials. Yet, the specific correlation between lignin’s structural features and the properties of the ensuing composite materials still needs to be further studied and systematized. Nevertheless, concerning the production of foams, Henry and coworkers [132] recently studied the suitability of nineteen unmodified lignins from various sources (hardwood, softwood, wheat straw, and corn stover) and isolation processes (kraft, soda, organosolv, sulfite, and enzymatic hydrolysis) as partial polyol (up to 30 wt.% of conventional polyol) in rigid PU/polyisocyanurate (PUR/PIR) foam formulations. The results showed that lignins with higher aliphatic and *p*-hydroxyphenyl contents, higher pH, and higher sodium, calcium, magnesium, and potassium contents performed well in PU foam preparation and testing.

Although it is possible to produce PUs with acceptable performance using formulations with up to 30 wt.% of lignin, this does not mean that lignin is incorporated into the final PU as cross-linker. An alternative to ensure the incorporation of lignin as cross-linker is its chemical modification via acid liquefaction and oxyalkylation, among others, as discussed in Section 4, which affords polyether polyol with up to 35 wt.% of lignin [9,10,57]. Recently, our group optimized the oxyalkylation of LignoBoost^TM^ kraft lignin with propylene carbonate (PC) to produce a crude LBP with suitable I_OH_ and viscosity to be used in the production of different PU products, such as rigid foams and adhesives [90]. In most studies, the lignin-based polyether polyol obtained from oxyalkylation using PO [144,145] or cyclic carbonates [15,146] has been used for the production of rigid PUF for thermal insulation. For this type of application, one of the most important properties is the thermal conductivity, which depends strongly on the cellular structure of the foam. The same is true for the mechanical properties of PUF. For these reasons, the addition of the LBP, which can have a significant effect of the cellular structure of the foam, needs to be fine-tuned in order to obtain PUF that can compete with conventional PU foams available in the market. Duval et al. [146] limited the amount of crude LBPs up to 25% in the PUF formulation, which allows obtaining PUF with thermal conductivity of about 25 mW/m.K and more than 90% closed cells, suitable for the RPUF market. The study of Pinto et al. [144] reported the use of different amounts of LBPs from oxyalkylation with PO to produce RPUF with thermal conductivity values in the range of 37.2–49.0 mW/m·K and enhanced fire retardancy properties.

Worthy of notice is the fact that the number of studies investigating the properties of PUF derived from LBPs obtained by acid liquefaction are little explored compared to the use of LBP from oxyalkylation reaction. This may be due to the fact that liquefaction-derived polyols usually have high acid numbers and need to be neutralized by bases such as NAOH, which can add cost to the process and affect the foams formulations [17]. Nevertheless, in general, PUF obtained from an LBP by acid liquefaction showed good thermal stability and high mechanical properties [89,147,148].

Gouveia et al. [149] prepared PU adhesives for wood bonding from kraft lignin oxyalkylated under mild conditions with PO. Recently, the same group prepared PU adhesives by mixing castor oil with up to 30% of LBPs oxyalkylated with PC, where the use of LBPs improved the Young’s modulus and adhesion [150]. So far, this is the only study that has reported the use of lignin oxyalkylated with cyclic carbonate to produce PU adhesives.

As for any other polyol, the use of modified or unmodified lignin in the production of PUs requires the optimization of several parameters, such as NCO/OH ratio and catalyst amount, to yield materials with suitable properties. In fact, it is crucial to compare the properties of lignin-derived PUs with those of conventional PUs to assess how significant candidate LBPs are to replace petroleum-based polyols. Nevertheless, prior to that, the properties of LBPs have to be determined, namely, the viscosity, MW, and I_OH_, to ensure they fall within the ranges previously discussed in Section 4.3.

A recent review by Peyrton and Avérous [151] compiled the main bio-based components used in PUF formulations, discussing their characteristics (including those of lignins and LBPs) and the corresponding relationship with the morphologies and properties of the resulting PUFs. An example is the viscosity of the polyol: higher values of viscosity tend to lead to smaller cell size of PUFs. However, if the viscosity is too high, coarser and less uniform cells will be obtained. Moderate viscosity values (5–25 Pa.s) are desirable to obtain homogeneous and small cell sizes, which can improve the thermal conductivity and mechanical properties [113,151]. In turn, the I_OH_ of polyol is correlated with the crosslink density: higher values of I_OH_ result in higher extent of crosslinking and thus higher density of PUF. A study by Gondaliya and Nejad [107] showed that foam properties such as density and compression force deflection were positively correlated with the OH content of lignin. The molecular structure and also the MW of lignins and LBPs affect the mechanical and thermal properties of PUF. For example, LBP obtained from oxyalkylation generates homopolymers, as discussed in Section 4. This homopolymer does not necessarily need to be removed, but these small polyols can increase the flexibility of the polymer network and thus decrease the compression strength as well as the thermal stability [145,152]. As opposed to the number of contributions regarding the preparation of foams, no publication has been found that systematizes the characteristics of LBPs required to prepare lignin-derived elastomers, adhesives, or films. In fact, even though Ma et al. [134] also reviewed the use of lignin in the preparation of foams and elastomers, despite the plethora of interesting and innovative examples, including some discussion regarding the role of some structural features of lignin, such as the ability to establish hydrogen bonds and *π* − *π* interactions to achieve, for example, self-healing elastomers, no systematic guidelines were provided. Nevertheless, what can be concluded from the analyses of several papers is that the effects of lignin or of LBPs have a significant impact on the glass transition (T_g_) of the resulting PU, thus affecting its performance. This is particularly relevant in the case of adhesives. On the other hand, lower values of molecular weight tend to be associated with PU elastomers with higher mechanical strength and homogenous morphology.

In conclusion, several studies have shown that the properties of lignin have a profound impact on the resulting PU product performance, e.g., foams, elastomers, coatings, and films, regardless of the lignin source and isolation process [132,133,134]. Nevertheless, with the exception of a few examples, the exact role of the structural features of lignin during the reaction with isocyanates is hardly discussed. Instead, the mixture of unmodified lignin with other polyols or of LBPs is treated globally as the polyol component of the PU. Yet, with the advancement of fractionation methodologies, a better understanding of the role of structural features of this renewable OH-rich aromatic material will certainly become clearer and bring further insights.

## 6. Lignin as a Building Block to Synthesize Non-Isocyanate Polyurethane (NIPU)

The industrial synthesis of isocyanates involves the reaction between primary amines and phosgene at high temperatures (100–200 °C). The latter is a highly toxic gas, produced by the reaction between carbon monoxide and chlorine gas. In turn, diisocyanates cause acute adverse health effects, such as irritation of the respiratory tract, eyes, and skin, being a major cause of occupational asthma in workers employed by the polyurethane industry [153,154,155,156,157]. Moreover, the two most widely used isocyanates in the PU industry, MDI (methylene diphenyl diisocyanate) and TDI (toluene diisocyanate), are highly reactive chemicals that bind to DNA and are probably genotoxic [158]. Furthermore, various household PU products, such as mattresses, pillows, cushion packaging, and insulating materials in building construction, among others, exhibit detrimental environmental impact on aquatic life, soil health, plants, and humans due to the presence of toxic components such as isocyanates, flame retardants, and amine-based catalysts [159]. Additionally, some compounds, such as carbon dioxide, carbon monoxide, hydrogen cyanide, acetaldehyde, and methanol, are released when PU products are burned and/or landfilled at their end-life, contributing to the greenhouse effect and having toxic effects on human health [160,161]. All these reported issues led the European Union to adopt the regulation where it was proposed to reduce the content of isocyanate, with the main goal being to ban its use in the future [162]. Therefore, the development of safe processes and products to replace hazardous isocyanate is increasing, which in the literature are described as non-isocyanate polyurethane (NIPU).

**Figure 10:**
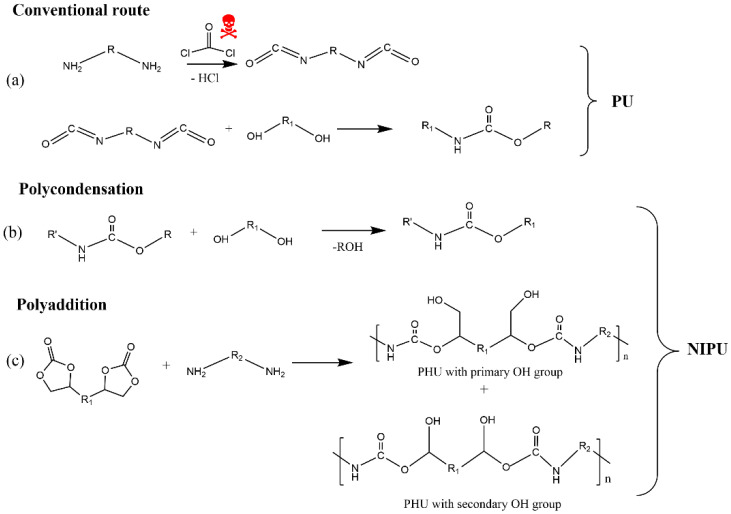
Chemical routes to produce PU by (**a**) conventional route, NIPU by (**b**) polycondensation, and (**c**) polyaddition.

There are a few routes described in the literature to synthetize NIPU, but the main ones are the rearrangement of acyl azide, the ring-opening polymerization of cyclic carbamates, the polycondensation (or transurethanization) between polycarbamate and polyol, and the polyaddition between bis-cyclic carbonate and diamines. Recently, some review works [160,163,164,165] dedicated to exploring the synthesis of NIPU were published, which identified that the most promising routes to produce NIPU are polycondensation and polyaddition (Figure 10).

The polycondensation reaction between carbamate monomers (bis-alkylcarbamates or bis-hydroxyalkylcarbamate) and a polyol affording urethane linkages have similar features compared to conventional PU. However, the polycondensation between bis-alkyl carbamates and polyol requires longer reaction times (over 24 h), higher temperatures (100–200 °C), and involves the formation of low-molecular-weight byproducts (i.e., methanol). Altogether, this has limited the extension of polycondensation on a commercial scale [166].

Another approach to obtaining NIPU involves the polyaddition of di- or polyamines to bis-cyclic carbonate, yielding poly(hydroxy urethane)s (PHUs). The most common cyclic carbonates used to synthetize PHUs are five-membered cyclic carbonates, such as EC, PC, GC, and VEC (mentioned in Section 4.1), and are already commercially available. Nowadays, the main route used to produce these cyclic carbonates is the carboxylation of epoxides, which is a less environmentally aggressive approach when compared to the previous synthetic route involving phosgene. With regard to the amines, they can be obtained from commercially available products such as ethylenediamine, hexamethylenediamine, tris(2-aminoethyl)amine, etc. [167]. The aminolysis of cyclic carbonates can be carried out under mild conditions from room temperature up to 120 °C in a solvent, with the most used solvents being DMSO and DMF [163,168]. Furthermore, the polymerization reaction does not release volatile organic compounds (VOCs) and the product obtained is non-moisture-sensitive, which is an advantage for coatings applications [169]. In general, this type of NIPU shows improvements regarding the thermal stability and chemical resistance to nonpolar solvents over conventional polyurethanes [170].

Beyond the strategies using phosgene-free routes to replace isocyanates, renewable resources have been extensively investigated to produce NIPU to obtain safe and green products. Recently, interesting reviews were published, mainly focused on the synthesis of intermediate cyclic carbonates derived from renewable resources and their reaction with polyamines to produce bio-based NIPU [21,171,172]. However, to the best of our knowledge, the direct use of renewable resources to synthetize NIPU has not been reported yet. The exploitation of renewable resources to produce NIPU has been focused on the synthesis of intermediate cyclic carbonates using feedstocks that come from three types of biomass: (1) vegetable oils and fat, (2) wood, and (3) starch and sugars. Each type of biomass has inherent features that will impact the final properties of NIPU as well as the chemical route to obtain the ensuing bio-based cyclic carbonate. Whilst, in general, vegetable oils provide the soft segments to the polymer [173,174], aromatic compounds such as lignin, tannins, and vanillin provide the hard segments responsible for the thermal stability and rigidity of the final product [175,176]. Furthermore, the chemical modification of biomass to obtain cyclic carbonates is different depending on the type of biomass used. Cyclic carbonates can be obtained via the epoxidation of vegetable oils and aromatic compounds, followed by carbonation with CO_2_ [175,177], whereas cyclic carbonates from sugars derivatives are obtained by direct carbonation with CO_2_ or dimethyl carbonate (DMC) [178,179].

Among all biomass, lignin is one of the natural polymers with higher potential for applications in polymeric materials, since it does not compromise the food supply and is generated in large amounts by the pulp and paper industry as a byproduct [38,180]. There is a substantial number of reports in the literature on the use of lignin, both unmodified and chemically modified, to produce bio-based PUs, some of which are already discussed in this review [46,47,95,132,181]. Nevertheless, every year, efforts to replace isocyanates are increasing, as well as the use of lignin or its derivatives to produce bio-based NIPU, since PU and NIPU from vegetable oils often show poor thermal and mechanical properties [182,183]. Vanillin and syringaresinol are aromatic compounds that have been used as a building block to develop several polymers, including NIPU, where, due to their aromatic structure, they may improve their thermomechanical properties [184,185]. The cyclic carbonates were obtained in two steps: (1) the glycidylation reaction between these aromatic compounds and epichlorohydrin; (2) the carbonation reaction between glycidyl ethers and CO_2_ [186]. Although vanillin and syringaresinol can be obtained by the depolymerization of lignin, the technology requires harsh oxidative or reductive conditions, which also lead to a high cost of the process [187].

An alternative to the isolation of specific aromatic compounds from lignin is its use as a macromonomer in the synthesis of NIPU. In actuality, Leet et al. [175] were the first research group to use lignin in the synthesis of NIPU, but not as a macromonomer. In this work, commercial epoxidized soybean oil (ESBO) was first carbonated with CO_2_ and then reacted with a coupling agent, 3-aminopropyltriethoxysilane, to form urethane bridges. This was followed by the addition of lignin whose OH groups reacted with the silane moieties, providing the hard segments of NIPU and subsequent increase of tensile strength. This bio-based NIPU achieved a high percentage of biomass in the formulation (85%). Salanti et al. [188] was the first group to use lignin as a macromonomer in the synthesis of NIPU. First, soda lignin was epoxidized with epichlorohydrin, and then the insertion of CO_2_ into the epoxidized lignin was carried out, at 20 bars for 20 h at 80 °C using KI (potassium iodide) as catalyst, to yield the cyclic carbonate. However, this strategy requires a high amount of epichlorohydrin, which is flammable and toxic. Furthermore, the insertion of CO_2_ into epoxidized lignin requires high pressure (20–40 bar), which can be challenging when scaling up the process. In another work reported by this group, lignin acted as cross-linker in the preparation of PHUs. This approach involved the reaction of cyclocarbonated lignin with 1,12-diaminododecane (chain extender) to prepare thermoset resins. The reactions were carried out in the presence of poly(ethylene glycol) bis-cyclic carbonate whose soft segment allowed tuning the resin properties [189].

**Figure 11:**
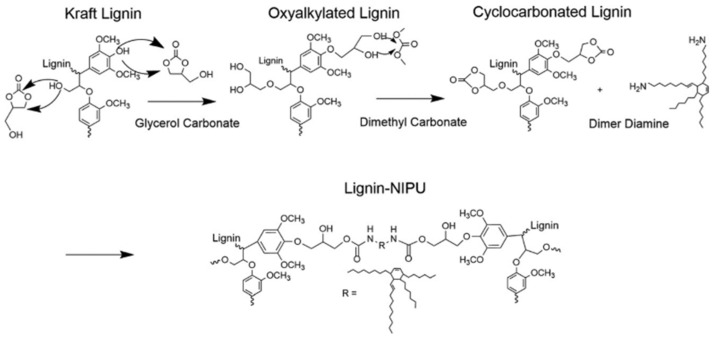
Strategy for the functionalization of kraft lignin to produce NIPU [192].

A novel strategy was developed by Kühnel et al. [190] to produce cyclocarbonated lignin as a building block to produce NIPU. The procedure was performed in two steps: (1) organosolv lignin was oxyalkylated using glycerol carbonate as the cyclic carbonate, affording a lignin-polyether with diol functionalities; (2) the oxyalkylated lignin was transesterified with dimethyl carbonate (DMC) under mild conditions (75 °C, 6 h, K_2_CO_3_ catalyst). Following this methodology, cyclocarbonated lignin can be obtained in high yield and the coproducts generated by oxyalkylation (CO_2_) and transesterification (methanol and ethylene glycol) can be recycled to produce DMC. Recently, lignosulfonate was derivatized following this approach, and the NIPU was successfully synthesized [191]. Due to the special properties of lignosulfonates, namely, water solubility and relatively high hydrophilicity, the lignosulfonate-derived NIPU had different material properties and application areas, as they present characteristics that conventional lignin-derived PUs do not have, such as compatibility with water and solvent, hydrogel formation, and swelling. Following the same approach, kraft lignin was used to produce NIPU (Figure 11) [192]. In addition, poly(methylhydrosiloxane) was used with the diamine and cyclocarbonated lignin to produce, for the first time, an NIPU foam with shape memory capacity. The authors emphasized that the innovation of this approach is the synthesis of cyclic carbonate precursors that can be compatibilized with a biobased curing agent (diamine). So far, it seems that this route is one of the most promising to derivatize lignin for NIPU synthesis.

Kraft lignin powder was also reacted directly with DMC and then with a diamine (e.g., HDMA) to yield NIPU. The products obtained were tested as coatings on the surface of beech wood [193]. In the same line of research, Arias et al. [194] prepared NIPU bio-adhesives for wood panels, reacting kraft lignin, organosolv lignin, tannin, and soy protein with DMC, and the intermediate products were reacted with hexamethylenediamine (HDMA). The research group evaluated the environmental profiles of these novel adhesives using the life cycle assessment (LCA) methodology and compared them with formaldehyde-based resins. The results showed that these bio-adhesives have the potential to replace synthetic resins, as they have a lower damage score than phenol-formaldehyde resin. Very recently, Meng et al. [195] used an alternative pathway to produce NIPU from lignin, where lignin was aminated (via the Manich reaction) using diethylene-triamine and formaldehyde under acid conditions and subsequently reacted with bicyclic carbonates to yield NIPU. This novel bio-based NIPU showed good thermal stability and high tensile strength.

Although it is clear that lignin has high potential to be used as a macromonomer in the synthesis of cyclic carbonates or even in the direct production of NIPU, its application still requires much research to further tailor the process and properties of these novel cyclic carbonates and NIPU.

## 7. Technology Assessment

**Figure 12:**
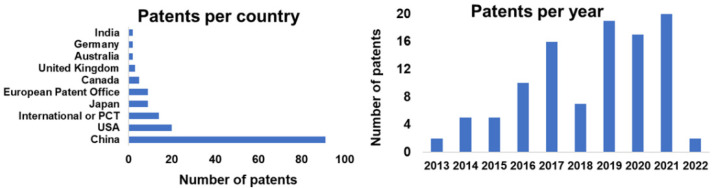
Summary of lignin-based polyurethane patents from 2013 to June 2022 obtained at the WIPO.

As previously mentioned, PUs are produced by the reaction of polyol and isocyanates; the use of polyols from renewable resources is already a reality and several companies are already commercializing bio-based polyols, as mentioned in the Introduction. Thus, lignin as a renewable resource rich in hydroxyl groups has a high potential as a raw material to produce PUs. The PUs market was worth around USD 73 billion in 2021, and the increase of demand for building insulation, in light of sustainability concerns, is expected to escalate the demand of bio-based PU, growing at 5.9% CAGR (compound annual growth rate) from 2021 to 2028 [6,196]. This growth has been accelerating the market demand for bio-based PUs, as well as the increase of the number of patents involving lignin in the production of PUs. Since 2013, 157 patents have been registered in the World Intellectual Property Organization (WIPO), with peaks in 2019 and 2021, using “lignin-based polyurethane” as keyword in the search. China is the dominant country for the application patents, due to the country’s greater demand in the market, followed by USA and the applications under the Patent Cooperation Treaty (PCT), as shown in Figure 12.

Mutual efforts have been made between pulp and chemical companies towards the development of lignin-based materials. For example, in 2016, the West Fraser Timber Company, a Canadian forestry company, started a plant in Hilton that can produce up to 30 tons per day of kraft lignin powder [52]. This company, in collaboration with Hexion S.A, a giant US corporation of the chemical sector, especially in the production of resins, have been producing lignin-based resins for plywood [197]. In 2017, Hexion filed an international patent regarding the synthesis of alkoxylated lignin to produce PUs, claiming a methodology that combines polyhydric alcohol and cyclic carbonates in the same process [16]. Another example is the case of FPInnovations who, in 2018, patented a process to produce bio-based polyols and phenols using kraft and hydrolyzed lignin for further production of lignin-based PUF and epoxy resins. The process consists of the depolymerization of lignin using polyhydric alcohols, which is followed by the oxyalkylation of lignin with alkylene oxides such propylene oxide and ethylene oxide [198]. The Enerlab 2000 company, using lignin supplied by the West Fraser Timber Company, developed the IsoLignin^®^ technology, which was patented in 2015 and is already commercially available. This technology allows the direct use of large quantities of lignin (up to 25 wt.% lignin content) in PUF, without requiring any chemical transformation [199,200]. This product has been used for foam insulation and insulating panels’ applications. Regarding the use of lignin to produce NIPU, research is at an early stage; however, promising results have already shown the potential of lignin as a precursor of NIPU. Recently, a patent from 2021 disclosed a bio-based polymer and methods for preparing NIPU, involving the preparation of a cyclocarbonated polymer from LBPs or other aromatic or cyclic bio-based polyols [201].

LBPs and PUs have high potential to compete with other bio-based polyols from vegetable oils (soybean, castor, and palm oils) since the market of bio-based PU is growing. However, intense work is still needed to answer several questions, for instance, issues related to the regulation of the content of bio-based chemicals in the final product, and whether the cost of chemical modification is profitable, or at least viable, not to mention policy implications. In this way, there must be a great synergy between the academy, producers, and consumers of lignin that, besides developing and registering new technologies, should answer these emerging questions.

Despite the fact that the use of lignin in the production of LBPs or the production of a precursor of NIPUs can be considered a promising raw material to produce renewable and sustainable polymers, some studies using the life cycle assessment (LCA) methodology proved that the use of renewable materials cannot guarantee better environmental performances when compared to fossil resources [202,203]. In general, LCA studies of lignin-based products have shown better environmental performance compared to products from petroleum resources [204,205,206,207]. However, the assessment of the environmental impact of lignin-based products by LCA can be a challenge, since almost all LCA studies were made from data obtained at the laboratory scale and from modeling processes. Besides that, in most cases, the LCA methodology applied is different for the same polymer in the same scope, which implicates huge differences in the results (200–400%) [208,209]. Nevertheless, LCA is still a great tool for labeling a bio-based product as sustainable if the methods of LCA are very carefully selected based on the goal and scope of the study.

Finally, the commercialization of polyurethane products from lignin can only become a reality after the biorefinery concept is well established in the pulp and paper industry, especially regarding the lignin extraction process. So far, 22 companies were identified that are commercially producing lignin (amounts above 5000 tons per year), namely, in North America and Nordic countries [52]. Countries that are important producers of pulp, such as Brazil, are also investing in the biorefinery concept; kraft lignin from the LignoBoost™ process is already a reality, with a capacity of 20,000 tons per year at Limeira mill, São Paulo (Suzano S.A.), and Klabin S.A. has announced a pilot-scale facility pilot plant at Telêmaco Borba, Paraná, with a capacity of one ton per day in 2020 [52]. Very recently, the Valmet company announced delivery of the LignoBoost plant to the Mercer Rosenthal pulp mill in Germany, with a capacity of around 350 tons per year, and the extracted lignin will be used for developing various bio-based materials [210].

## 8. Concluding Remarks

On the one hand, technical lignin is a byproduct of the pulp and paper industry that consists of natural aromatic polymers with high carbon content and is a valuable energy source. For example, in the kraft pulp industry, lignin is used entirely for energy recovery. On the other hand, technical lignin has great potential to replace, at least partially, petroleum-based polyols used in the formulation of PU products. Yet, at the moment, to obtain an LBP that is competitive with petroleum-based polyols in terms of characteristics and price is still a challenge. Indeed, there is an old saying among the lignin experts: “you can make anything out of lignin, except money”. This is due to the difficulties involved in obtaining technical lignin with homogenous reproducible characteristics, the cost to convert the solid lignin in a liquid polyol or other chemicals, and the poor compatibility of lignin with many synthetic polymers. However, the pulp and paper industry, in cooperation with the chemical industry, have shown that it is possible to earn money from lignin, otherwise companies would not be investing in it. Although LBPs are not yet on the market, polyurethane foams with added lignin (20–25 wt.%) in the formulations are already a reality in the market. Furthermore, developments regarding the depolymerization and fractionation of lignin into well-defined oligomonomers are required to avoid the need for chemical modification. Indeed, these developments will also contribute to a better understanding of the relevance of the structural features of lignin during the reaction with isocyanates and/or other chemicals, as well as their exact impact on the properties of the ensuing PUs. Likewise, in-depth studies regarding the dissolution of lignin into polyols, especially those obtained from renewable resources, show that this is also a challenge, and an opportunity exists to make the use of this rich and versatile material economically and environmentally viable. As final recommendations on the development of lignin-based polyurethanes, researchers and industry should pay attention to LCA studies and technical–economic assessments. These studies are of paramount importance to find solutions regarding current challenges, such as technology scaling up for lignins and LBPs, high process and product cost, and different environmental and social impacts, as well as regulatory and policy implications.

Beyond the development of bio-based polyols to produce polyurethanes, significant efforts have also been made to replace the toxic isocyanate, developing non-isocyanate polyurethane. The main chemical route employed is the reaction between cyclic carbonates and diamines. Moreover, in recent years, lignin has been studied as a building block in the synthesis of cyclic carbonates for the development of new isocyanate-free polyurethanes. It is evident that there are still many challenges to be overcome, but what is expected is that in the future, technical lignin will be used as a raw material for the production of polymeric products such as polyurethanes, and not just burnt for energy, thus contributing to a more sustainable society.

## Figures and Tables

**Table 3:** Some properties of PO and PC, adapted from [63].

Properties	PO	PC
Boiling point, °C	34	242
Flash point, °C	−37	135
Vapor pressure, hPa at 20 °C	588	0.04

**Table 4:** Summary of operating conditions and some characteristics of oxyalkylated lignin according to the literature [9,10,15,55,59,65,75].

Parameters	Range of Conditions	
	Alkylene Oxides	Cyclic Carbonates
Temperature, °C	140–235	100–200
Pressure, bar	6–20	1
Time, hour	0.25–15	0.5–24
Type of catalyst	KOH, NaOH, tertiary amines	K_2_CO_3_, KOH, tertiary amines
Catalyst/lignin ratio, wt. %	1–10	0.25–10
Amount of reagent, eq. molar	1–10 *	5–50
Formation of homopolymer	High	Low–medium
I_OH_, mg KOH/g	82–445	164–569

* wt.%.

**Table 5:** Summary of operating conditions of acid liquefaction of lignin using H_2_SO_4_, and their effect on the formation of solid residue [13,13,19,82,84,85,86].

Parameters	Range of Conditions	Solid Residue *
Temperature, °C	130–180	++
Time, hour	1–3	+++
Solvents	PEG 400, EG, glycerol, crude glycerol, or a combination	++
Weight ratio of solvent/lignin	3:1–9:1	+++
Catalyst loading (H_2_SO_4_), %	1–6	+++

* Qualitative evaluation established by comparison of the articles considered, where ++ and +++ refer to medium and high impact of parameters on the formation of solid residue, respectively.

## Data Availability

Not applicable.

## Abbreviations

EC	ethylene carbonate
EO	ethylene oxide
GC	glycerol carbonate
LBP	lignin-based polyol
PUF	polyurethane foam
PC	propylene carbonate
PO	propylene oxide
PUs	polyurethanes
NIPU	non-isocyanate polyurethane
VEC	vinyl ethylene carbonate
